# Sociality Mental Modes Modulate the Processing of Advice-Giving: An Event-Related Potentials Study

**DOI:** 10.3389/fpsyg.2018.00042

**Published:** 2018-02-06

**Authors:** Jin Li, Youlong Zhan, Wei Fan, Lei Liu, Mei Li, Yu Sun, Yiping Zhong

**Affiliations:** ^1^Department of Psychology, Hunan Normal University, Changsha, China; ^2^Cognition and Human Behavior Key Laboratory of Hunan Province, Changsha, China; ^3^Research Center of Brain and Cognitive Neuroscience, Liaoning Normal University, Dalian, China

**Keywords:** sociality mental mode, advice-giving, event-related potentials (ERPs), N1, P300

## Abstract

People have different motivations to get along with others in different sociality mental modes (i.e., communal mode and market mode), which might affect social decision-making. The present study examined how these two types of sociality mental modes affect the processing of advice-giving using the event-related potentials (ERPs). After primed with the communal mode and market mode, participants were instructed to decide whether or not give an advice (profitable or damnous) to a stranger without any feedback. The behavioral results showed that participants preferred to give the profitable advice to the stranger more slowly compared with the damnous advice, but this difference was only observed in the market mode condition. The ERP results indicated that participants demonstrated more negative N1 amplitude for the damnous advice compared with the profitable advice, and larger P300 was elicited in the market mode relative to both the communal mode and the control group. More importantly, participants in the market mode demonstrated larger P300 for the profitable advice than the damnous advice, whereas this difference was not observed at the communal mode and the control group. These findings are consistent with the dual-process system during decision-making and suggest that market mode may lead to deliberate calculation for costs and benefits when giving the profitable advice to others.

## Introduction

People satisfy their needs in mainly two ways: via close communal ties or dealings with others in a marketplace (Gasiorowska et al., 2016). Specifically, people in different sociality modes (i.e., communal and market modes) interact with others in distinct manners, thus sociality mental modes might influence the social interactions (Fiske, 1992; Clark and Mils, 1993), even influence the advice-giving. Advice-giving is a common social decision-making in daily life, which is described as advisor attempts to aid the advisee to solve their problem (Lippitt, 1959; Mobbs et al., 2015). If advisor act in different modes, the processing of advice-giving in the communal mode and market mode might be different. However, relatively little is known about the underlying neural mechanisms. In the present study, we asked participants in different sociality mental modes to decide whether or not give the profitable or damnous advice to a stranger without any feedback, during which their ERPs components were recorded. Our aim is to explore how these two types of sociality mental modes affect the processing of advice-giving.

Sociality mental modes affect attendant behaviors, mindsets, and motives (Fiske, 1992; Clark and Mils, 1993). The sociality mental modes mainly can be classified into two types: communal mode and market mode. In communal mode, people perform a general concern for others based on reputation (Clark and Mils, 1993). Meanwhile, they also readily share resources with others regardless of the personal costs and benefits (Baumeister and Leary, 1995). Instead, in the market mode, all other relationships are organized in terms of cost-benefit ratios (Fiske, 1992), and the market mode underlies cost-benefit analyses, in that a person consider what he would receive in return before conducting behaviors (Vohs et al., 2008). Experimental evidence showed that the sociality mental modes could be elicited by different cues. In particular, money is the most typical cue of market mode and the presence of banknotes can elicit the market-pricing orientation (Vohs et al., 2008; Caruso et al., 2013; Gasiorowska et al., 2016). A large body of experimental evidence showed that money cues could elicit the market mode (Fiske, 1992; Heyman and Ariely, 2004; Lea and Webley, 2006; Zhou et al., 2009). Studies showed that after participants were exposured to images of banknote, people were inclined to focus on the personal costs and benefits and people moved the social system to confer benefits (Vohs et al., 2008; Zhou et al., 2009; Gasiorowska and Hełka, 2012; Sarial-Abi and Vohs, 2012; Trzcińska and Sekścińska, 2016). Together, these studies showed that by priming the money cues, the person would interact with others through calculating the ratio between what one’s costs and what one is likely to benefit (i.e., market mode) (Caruso et al., 2013; Mead and Stuppy, 2014; Savani et al., 2016). Alternatively, eye cues, especially eye-gaze cues, are the important cues of communal mode (Emery, 2000). Researches have confirmed that cues of eye-gaze could elicit the communal mode, and people who primed with eye cues more inclined to concern others and provide benefits to other individuals without concerning the personal costs in social behaviors such as donation, civic behavior or decision making (Haley and Fessler, 2005; Bateson et al., 2006; Ekström, 2012; Powell et al., 2012; Fathi et al., 2014). Overall, the distinction of processing of behavior in two sociality mental modes was confirmed by previous research (Clark and Jordan, 2002). In particular, when people perform in the market mode, they will concern personal analytical processes instead of others’, and help the other according to reciprocity (Heyman and Ariely, 2004; Gasiorowska et al., 2016). Simon (1993) proposed that persons low in concern for others were inclined to engage in more deliberate computations involving personal costs and benefits. In contrast, people execute in the communal mode have a high concern for others (Fiske, 1992), and are likely to attach less importance to the personal costs and benefits (Korsgaard et al., 2007), since they concern for others based on the motivation of reputation-seeking (Baumeister and Leary, 1995; Heyman and Ariely, 2004). Thus, sociality mental modes will exert an influence on social behaviors by affecting the motivations.

Furthermore, sociality mental modes also might modulate the processing of social decision-making. Fiske (1992) proposed that individuals in different sociality mental modes had the different strategies to make decisions in social life. For example, Gasiorowska and Hełka (2012) found that individuals in the market mode decided to transfer smaller money to others in the dictator game. Instead, Nettle et al. (2013) confirmed that in the communal mode, the decisions of giving money to others increased. Thus, two sociality mental modes affect the processing of social decision-making differently.

Actually, many social decisions that we make are only on behalf of other people (Tunney and Ziegler, 2015). A common type of these decisions is advice-giving. When advisors have a useful information and they decide to give the advice others or not (especially the advisor is the unique one to give advice without the third party), thus they consider themselves as the decision makers for others (Jonas et al., 2005; Garvin and Margolis, 2015). Generally speaking, people prefer to give profitable information to others, even others are strangers (Rosen and Tesser, 1970), since advice-giving is an attempt to manipulate what others think about themselves (i.e., reputation seeking) (Jonas et al., 2005; Patt et al., 2006; Izuma, 2012). Giving good advice rather than poor advice affords us opportunities for reputation enhancement since others would believe that we are knowledgeable, trustworthy or indispensable (Jonas et al., 2005; Helm and Salminen, 2010; Mobbs et al., 2015). Prior studies showed that the motivation of such social decision-making could be mainly affected by different sociality mental modes (Fiske, 1992; Clark and Mils, 1993; Heyman and Ariely, 2004; Gasiorowska et al., 2016). When people make decisions in different sociality mental modes, how the motivations underlie the advice-giving are affected? In the present study, we experimentally primed two sociality mental modes and conducted an event-related potentials (ERPs) study to investigate how different types of sociality mental modes affect the processing of advice-giving behavior. As mentioned above, advice-giving is considered as a usually example in social decision-making behavior. Thus, we created an advice-giving situation (the advisor was the unique one the advisee seeks for advice), and the participants (advisors) had a useful information, and asked him to decide whether or not to give the information to other without any feedback. In real life, we are actually not sure whether the advisee takes the advice immediately. Thus we mainly focus on the advice giving stage regardless of how the advisee makes the final decision. ERPs have been widely used to study social decision-making, with its high temporal resolution enabling detailed insights into the temporal course of decision-making (Gangl et al., 2017). For example, N1, which is a negative wave peaking approximately 100–150 ms after stimulus onset. This ERP component represents the selective attention processing at the early stage during decision-making task (Wang et al., 2001; Gui et al., 2016). Some studies have revealed that brain regions such as the central and parietal region was recruited during the early stages of valence-related information processing (Gui et al., 2016). The higher N1 amplitude suggests that individuals pay more attention on the valence of choice item very early during the decision process (Wichary et al., 2017). Crucially, the amplitude variation of P300, which is a positive wave peaking roughly 300–500 ms at central and parietal electrodes (Gu et al., 2011; Wang et al., 2017b), is a typical ERP component in social decision-making. More increased P300 amplitudes are related with a greater change in evaluative stimulus categorization (Cacioppo et al., 1993; Ito et al., 1998; Gangl et al., 2017) and greater mental resource demanded (Polich, 2007).

Therefore, we expected that two priming cues of two sociality mental modes could modulate the motivations of the advice-giving. After primed with money cues, people would perform in the market mode, when they had a profitable advice to make others earn, they might take more time in giving it to others without immediate return. In contrast, with the eye-gaze cues priming, people behaved in the communal mode, when they had a profitable advice, they preferred to give it to others and regardless of the direct return and would not take much time to engage in personal cost and benefit calculations deliberately. Moreover, at a neural level, as mentioned above, compared with communal mode, market mode demands more deliberate mental processing, thus we predicted that P300 amplitudes would be larger in the market mode during deciding to give the advice to others compared with which in the communal mode.

## Materials and Methods

### Participants and Design

Eighty-one undergraduates (39 males, *M*_age_ = 20.6 years old, *SD* = 3.73) participated in this experiment, which is a 2 (Attributes of advice: profitable vs. damnous) × 3 (Types of sociality mental modes: Market mode vs. Communal mode vs. Control group) mixed design with repeated measures on the first factor. 28 participants were assigned to the Market mode; 25 participants were assigned to the Communal mode; 28 participants were assigned to the Control group. All participants were right-handed, had normal or corrected-to-normal vision and reported no history of traumatic brain injury, brain surgery, and mental or neurological diseases. Prior to testing, each participant signed an informed consent form. The Ethics Committee of Hunan Normal University approved this study.

### Stimuli

The size of all stimuli were 207 pixels × 155 pixels. All stimuli were 3 cm wide and 5 cm high. The value of luminance and contrast were the same across the images. The stimuli were displayed on 17-inch cathode-ray tube (CRT) monitors with 75 Hz refresh rate. Participants were seated in a dim room, at a viewing distance of 75 cm, with the horizontal and vertical visual angles below 5°. The software package E-prime 2.0 (Psychological Software Tools, Pittsburgh, PA, United States) was used for stimuli presentation and data collection. All stimuli (cues) were presented on a background showed the bottom of the sea (without any other images).

#### Cues of Market Mode

Prior researches have shown that money was the symbol of market mode, by priming the images of banknotes, it would elicit the market mode (Vohs et al., 2008; Gasiorowska et al., 2016). Thus we selected the front image of RMB banknote (the denomination was 100 Yuan).

#### Cues of Communal Mode

Based on previous studies (Haley and Fessler, 2005; Bateson et al., 2006; Fathi et al., 2014), we selected the eye-gaze images as the cues to elicit the communal mode.

#### Stimuli of Control Group

Based on the previous study (Vohs et al., 2008), we chose the images of ordinary tropical fish as the stimuli for control group, since fish is a common animal in the sea bottom.

Prior to the main experiment, a rating study was conducted to evaluate and select priming stimuli. We asked 60 participants to rate familiarity degree and emotion arousal degree on a 5-point Likert scale, and conducted a repeated measures ANOVA on the degree of arousal and familiarity. The results showed that the familiarity of three types of stimuli was statistically equivalent, *F*(3,59) = 0.41, *p* > 0.05. There was also no difference in emotion arousal level, *F*(3,59) = 0.65, *p* > 0.05.

### Procedure

Before the experiment, participants were instructed to imagine being a role of the advisor. A person who you never know played a score investment game, but this person was unsure about whether to invest score, thus he seeks the unique advisor (you) for the advice. The advisor may have a profitable advice [i.e., advising the advisee to invest, and he is more likely (90%) to gain 10 scores, and the possibility is fixed] or a damnous advice [i.e., advising the advisee to invest, and he is more likely (90%) to lose 10 scores]. The task of participants was to only decide whether or not give the profitable/damnous advice (reflected in different colors) to the stranger without any payment. If the advisee takes this piece of advice (he doesn’t know the attribute of advice), he might follow your advice to invest his score to this game. Each trial consisted of two stages: the priming stage and the advice-giving stage.

#### Priming Stage

All participants were assigned to three experimental conditions randomly. In Market Mode condition, participants counted the money and answered whether the number of banknote images was more than 10; in Communal Mode condition, participants counted the pairs of eyes and answered whether the number of pairs of eyes-gaze images was more than 10; in control group condition, participants counted the number of tropical fish and answered whether the number of fish images was more than 10. In all three conditions (market mode, communal mode and control group), participants were seated in a comfortable chair in front of a computer screen.

**FIGURE 1:**
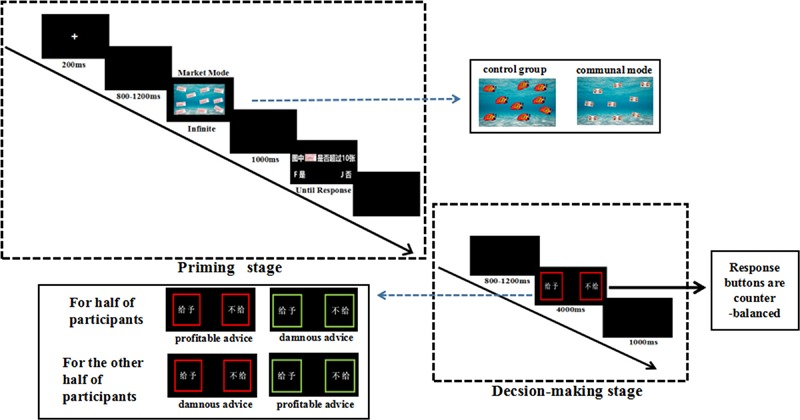
An illustration of a single trial in the advice-giving task. Each trial began with a fixation cross. Each participant gets different sociality mental modes operations. Then they were required to choose one of the boxes (giving or not giving) by pressing the corresponding key.

As shown in **Figure 1**, at the priming stage, each trial began with the presentation of a white cross (200 ms), followed by a blank screen (random duration of 800–1200 ms). Subsequently, three different priming stimuli were presented (2000 ms), followed by a blank screen (1000 ms), then appeared a screen asking whether the number of the stimuli was over 10 (no time limit). Then a blank screen was presented for 500 ms.

#### Advice-Giving Stage

After primed with different sociality mental modes, at the advice-giving stage, a blank screen was presented for a random duration of 800–1200 ms. Afterward, the advice type presented, and participants indicated whether or not they gave the profitable or damnous advice to other in this interface by pressing buttons (“F” or “J”; the response buttons are counterbalanced across participants) (the meaning of the color of frame are counterbalanced across participants: for 40 of participants, the red frame of the box represented the profitable advice, and the green frame of the box represented the damnous advice; and for other 41 participants, the color of two meanings was opposed). Then the blank screen was presented again for 1000 ms. Each condition contains 80 trials. After completed the experiment, each participant filled out the manipulation check items.

### Manipulation Check Measures

Previous studies suggested that individuals who were in the market mode expected to help others when they received or anticipated receiving a comparable benefit in exchange, and effort would increase with payment level. In contrast, individuals who behaved in the communal mode expected to help others out of a caring or concern for the others’ needs, and effort would be insensitive to payment level (Clark and Mils, 1993; Heyman and Ariely, 2004; Johnson and Grimm, 2010). Thus, we assessed the effectiveness of the manipulation with three items used in these previous works. Specifically, after manipulated with different experimental conditions, all participants answered three questions: (a) “Are you willing to help others with great effort without any payment?”; (b) “Are you willing to help others with great effort with receiving 10 RMB (i.e., a low payment)?”; (c) “Are you willing to help others with great effort with receiving 100 RMB (i.e., a medium payment)?” and they indicated from 1 (be unwilling to help at all) to 5 (be willing to help at all).

### ERP Acquisition and Analysis

**FIGURE 2:**
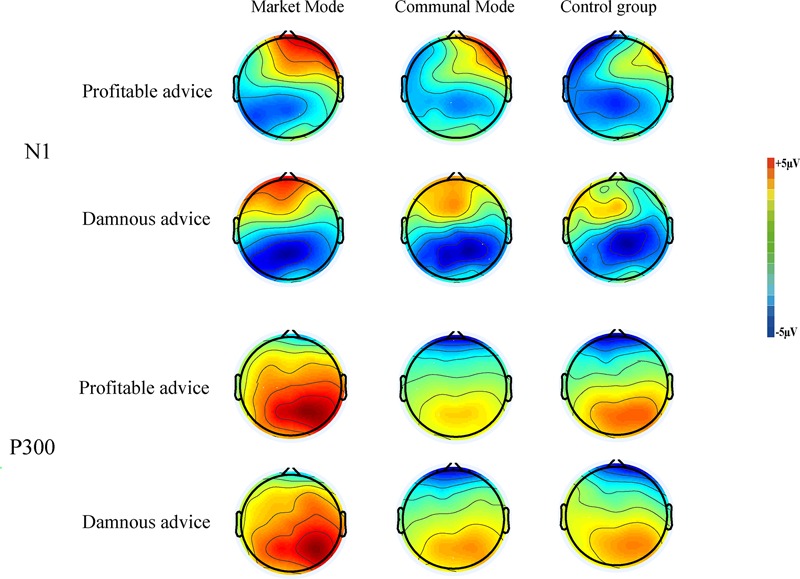
Topographical voltage distributions of the N1 (100–200 ms) and P300 (350–450 ms) for each conditions.

Electroencephalogram (EEG) was recorded from 64 scalp sites using tin electrodes mounted in an elastic cap, according to the International 10/20 EEG/ERP System (NeuroScan Inc., United States). The impedance at all recording sites was maintained below 5 kω. Eye movements were recorded from left supraorbital and infraorbital electrodes and electrodes were placed 1.5 cm lateral to the left and right external canthi. The EEG recording was continuously sampled at 500 Hz with a left mastoid reference and a forehead ground. EEG data were re-referenced to the average of the left and right mastoid, filtered with a 0.1–30 Hz bandpass filter. Independent component analysis algorithm was utilized to remove blinks and movement artifacts (Delorme and Makeig, 2004; Plöchl et al., 2012). We visually inspected the whole EEG data and removed trials containing high amplitude noise, such as large body movements related muscle activity potential, extrusive eye blinks and saccade-related artifacts, as well as other easily identifiable confounds such as sudden electrode drifts and jumps. Trials in which EEG voltages exceeded a threshold of ±75 μV were excluded from the analysis. Epochs were extracted from the continuous data files from 200 ms before to 800 ms after the onset of each decision interface presentation. Activity in the -200 ms to 0 ms time-window prior to the decision interface presentation served as the baseline for each ERP. ERPs were then derived by averaging the trials for each of conditions. Based on the evidence that N1 and P300 from central and parietal sites are more suitable as an index of decision-making (Polich, 2007; Carlson et al., 2015; Gui et al., 2016; Hu et al., 2017; Wang et al., 2017a,b) and the topographical distribution of each ERP components (**Figure 2**), we selected nine electrodes in central and parietal area of C3, Cz, C4, CP3, CPz, CP4, P3, Pz, and P4 for N1 and P300 (Hu et al., 2017). For the nine electrodes in central and parietal area, we measured mean amplitude in their corresponding time window (i.e., N1 within 100–200 ms and P300 within 350–450 ms), and these mean values were then averaged to produce our final N1 and P300 components (i.e., it was performed a mean value on the adjacent electrodes of interest). The statistical analysis was conducted using SPSS 20.0. The *p*-values for main and interaction effects were corrected using the Greenhouse–Geisser method for violations of the sphericity assumption, and Bonferroni corrections were used in cases of multiple comparisons.

## Results

### Manipulation Check

**FIGURE 3:**
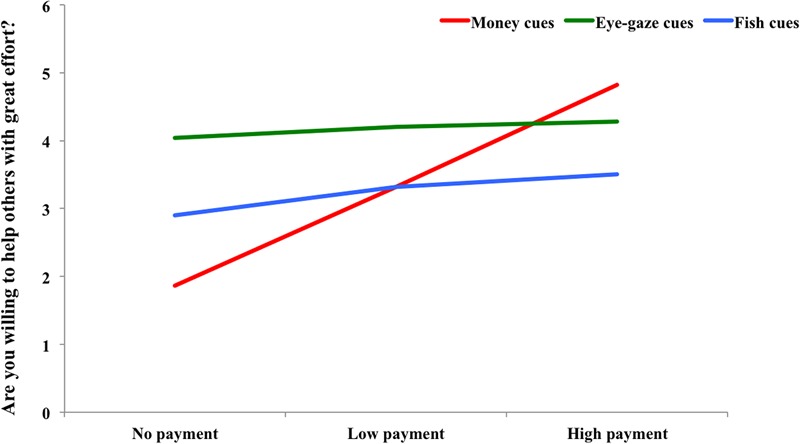
The line graph of the manipulation checks measure.

We examined the participants’ responses to the manipulation check to determine whether primed with different types of cues, they would indeed perform in accordance with the market mode or communal mode. For the degree of willing to help others, we conducted a 3 (Types of priming cues: money images vs. eye-gaze images vs. tropical fish images) × 3 (Benefits: helping without payment vs. helping with low payment vs. helping with high payment) mixed-model ANOVA with repeated measures on the second factor. As shown in **Figure 3**, there was a main effect of Types of priming cues, *F*(2,78) = 25.94, *p* < 0.001, $\eta_{p}^{2}$ = 0.40, and after primed with eye-gaze cues, participants were more likely to help others with effort (*M* = 4.17) than the participants who were primed with money cues (*M* = 3.33) and fish image cues (*M* = 3.24). And there was also a main effect of Cost, *F*(2,156) = 185.64, *p* < 0.001, $\eta_{p}^{2}$ = 0.70. If receiving the high payment, participants were more likely to help others with effort (*M* = 4.20) than received no payment (*M* = 2.93) and low payment (*M* = 3.61). There was a significant interaction effect of Types of cues and Cost, *F*(4,156) = 84.89, *p* < 0.001, $\eta_{p}^{2}$ = 0.69. The follow-up analyses showed that when primed with money cues, the degree of willingness to help with effort increased significantly along with the increase of payments (*p*s < 0.001); when primed with eye-gaze cues, there was no difference in the degree of willingness to help among three types of payments (*p*s > 0.05). In the control condition, when the participants would receive payments (whether high or low), they were more willing to help others than help others with no payments, *t*(27) = 4.50, *p* < 0.001 (low payment – no payment); *t*(27) = 4.69, *p* < 0.001(high payment – no payment), respectively. Therefore, after primed with money images or eye-gaze cues, the participants performed in accordance with the market or communal mode (Heyman and Ariely, 2004). Thus, the manipulation had the expected effect.

### Behavioral Data

#### Priming Stage

##### The accuracy and reaction times in priming task

There was no difference in RTs of counting the stimuli, *F*(2,78) = 0.32, *p* = 0.74. And there was also no difference in accuracy and reaction times in answering the number of cues at the priming stage among three experimental conditions [ACC: *F*(2,78) = 1.29, *p* = 0.28; RTs: *F*(2,78) = 0.61, *p* = 0.61].

#### Advice-Giving Stage

##### The proportion of choosing to give and the decision time

**FIGURE 4:**
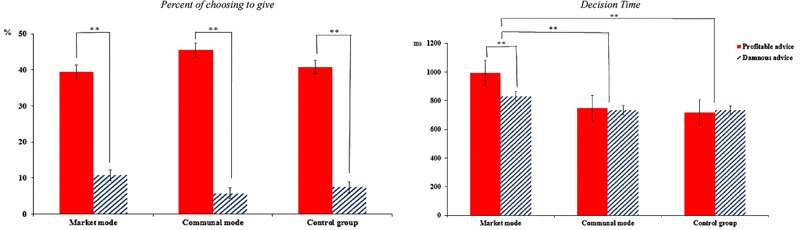
The proportion of choosing to give and the decision time for each condition. ^∗∗^*p* < 0.01.

The proportion of giving was submitted to a 2 (Attributes of advice: profitable vs. damnous) × 3 (Mode types: market mode vs. communal mode vs. control group) mixed-model ANOVA with repeated measures on the first factor. There was a significant main effect of Attributes of advice, *F*(1,78) = 671.20, *p* < 0.001, $\eta_{p}^{2}$ = 0.89. Compared with damnous advice (*M* = 12.7%), people preferred to give profitable advice to other (*M* = 41.0%). There were no other effects. And the decision time (DT) were also submitted to a 2 (Attributes of advice: profitable vs. damnous) × 3 (Mode types: market mode vs. communal mode vs. control group) mixed-model ANOVA. A significant main effect of Attributes of advice emerged, *F*(1,78) = 5.48, *p* = 0.022, $\eta_{p}^{2}$ = 0.10. Compared with profitable advice (*M* = 820.31 ms), people would take less time to decide when confronted with damnous advice (*M* = 764.99 ms). A significant main effect of mode types also emerged, *F*(2,78) = 15.20, *p* < 0.001, $\eta_{p}^{2}$ = 0.28. *Post hoc* analyses showed that compared with the market mode (*M* = 911.83 ms), people in communal mode (*M* = 740.99 ms) and control group (*M* = 725.14 ms) take less time to decide whether or not give the advice. There was a significant interaction between these two factors, *F*(2,78) = 5.51, *p* = 0.006, $\eta_{p}^{2}$ = 0.12. A simple effect analysis showed that in communal mode and the control group, there was not significant in DTs between decide whether or not give the profitable and damnous advice, *p*s > 0.05. In contrast, in the market mode, compared with the profitable advice, the participants would take less time to make the decision when confronted with the damnous advice, *F*(1,78) = 15.50, *p* < 0.001, $\eta_{p}^{2}$ = 0.32 (see **Figure 4**).

### ERP Data for Advice-Giving Stage

#### N1 (100–200 ms)

Mean N1 amplitude was analyzed using a 2 (Attributes of advice: profitable vs. damnous) × 3 (Mode types: market mode vs. communal mode vs. control group) mixed-model ANOVA with repeated measures on the first factor. We found the main effect of Attributes of advice was significant, *F*(1,78) = 15.45, *p* < 0.001, $\eta_{p}^{2}$ = 0.17. The damnous advice (*M* = -2.97 μV) elicited more negative amplitude than profitable advice (*M* = -2.32 μV). There were no other effects, *p*s > 0.05.

#### P300 (350–450 ms)

**FIGURE 5:**
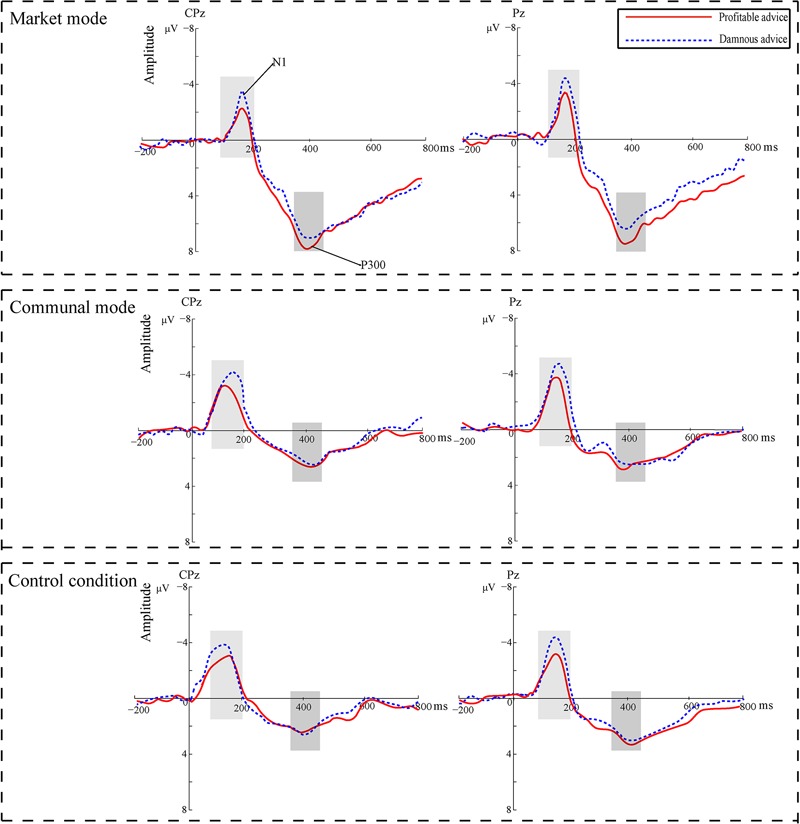
Grand-average ERP waveforms from the CPz and Pz electrode sites.

**FIGURE 6:**
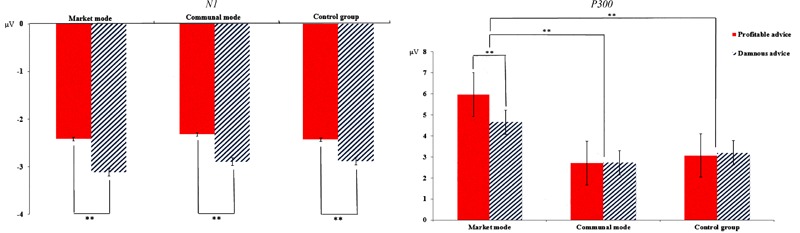
The mean value of the N1 and P300 amplitude for each condition. ^∗∗^*p* < 0.01.

We also entered the P300 mean amplitude within 350–450 ms into a mixed-model ANOVA with repeated measures on the first factor. We found a significant main effect of Attributes of advice, *F*(1,78) = 9.06, *p* = 0.004, $\eta_{p}^{2}$ = 0.10. A profitable advice (*M* = 3.92 μV) elicited larger P300 amplitudes than a damnous advice (*M* = 3.24 μV). A main effect of Mode types emerged, *F*(2,78) = 10. 44, *p* < 0.001, $\eta_{p}^{2}$ = 0.21. *Post hoc* analyses showed that the market mode (*M* = 5. 32 μV) elicited larger amplitudes than the communal mode (*M* = 2.72 μV) and control group (*M* = 3.14 μV), *t*(51) = 4.19, *p* < 0.001, *d* = 0.71; *t*(51) = 3.61, *p* = 0.002, *d* = 0.59. **Figure 5** shows grand-average ERP waveforms at the CPz and Pz electrode sites. **Figure 6** shows the bar graphs show the mean value of the N1 and P300 amplitude for each condition.

Importantly, we observed a significant interaction between Attributes of advice and Mode types, *F*(2,78) = 13.68, *p* < 0.001, $\eta_{p}^{2}$ = 0.26. A simple effect analysis showed that in communal mode and the control group, there was no significant in the amplitudes of P300 between decide whether or not give the profitable and damnous advice, *p*s > 0.05. In contrast, in the market mode, there was a significant difference between good advice and damnous advice, *F*(1,78) = 36.68, *p* < 0.001, $\eta_{p}^{2}$ = 0.47. Compared with the good advice (*M* = 5.97 μV), the damnous advice (*M* = 4.67 μV) elicited smaller amplitudes.

**FIGURE 7:**
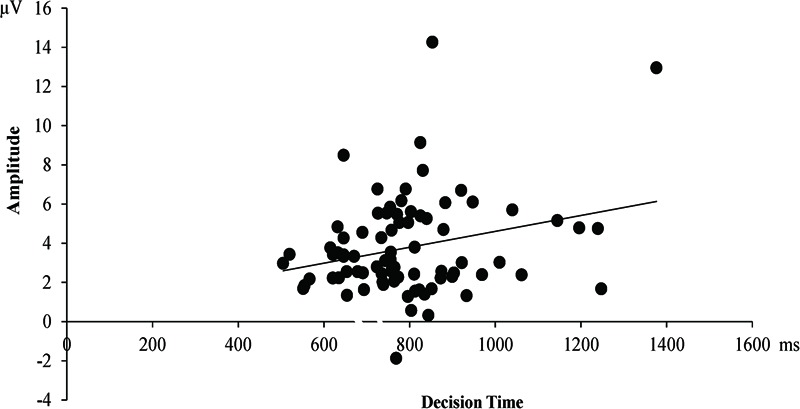
P300 amplitude (μV) in response for decision-making was positively correlated with the decision time.

In addition, we observed that a positive correlation between P300 amplitudes and the decision time, *r*(81) = 0.26, *p* = 0.018, suggesting that the greater P300 responses were associated with more response times for decision-making (see **Figure 7**).

## Discussion

The present study investigated the behavioral performance and temporal dynamics of processing of advice-giving in the two different sociality mental modes (i.e., market mode and communal mode). The results showed that, people preferred to give profitable advice to other across three conditions. In the market mode, compared with the profitable advice, the participants took less time to make the decision when confronted with the damnous advice. But in communal mode and the control group, there was not significant in DTs between profitable and damnous advice. At a neural level, the N1 was more negative for damnous advice compared with profitable advice. And the market mode elicited larger P300 amplitudes than the communal mode and control group. More importantly, compared with the good advice, the damnous advice elicited smaller P300 amplitudes in the market mode, while there was no such difference were observed in communal mode and control condition.

Consistent with our predictions, people are willing to give profitable advice to others, since the advice-giving is one attempt to seek the reputation (Izuma, 2012; Mobbs et al., 2015). On contrast, giving a poor advice can lead to emotions associated with doing interpersonal harm (e.g., guilt or embarrassment) (Cialdini et al., 1976; Bernstein et al., 2017). Furthermore, in the communal mode and the control group, the difference of response times for decision-making is not significant between profitable and damnous information. Conversely, participants in the market mode needed more time to decide whether or not to give the beneficial information to others compared with a piece of poor information. This can be due to different mental modes have different calculation of costs and benefits (Gasiorowska et al., 2016). Specifically, the costs-benefits calculation demands the mental operations. Communal mode demands relative little mental processing for personal costs and benefits. In market mode, people attached more importance on personal costs and benefits, and they might have a deliberate computation involving “Good costs deserve good personal benefits.” When participants decide to give a beneficial advice to others without any corresponding good immediate return (feedback), they would have a conflict in giving the positive information to others without any immediate benefits, thus the decision time become longer.

On the neuronal level, we found that the negative advice elicited the more negative amplitude of N1 than good advice. Wang et al. (2001) found that N1 component was related to early selective attention. Considerable research indicated that human brain was especially sensitive to negative information since the salient of valence, and that the negative information is preferentially processed relative to neutral and positive events (Huang and Luo, 2006; Yuan et al., 2007; Gui et al., 2016). The higher N1 amplitude suggests that individuals pay more attention on this choice item at the early stage during the decision process (Wichary et al., 2017). Thus, the enhance N1 reflected that the negative information attracted people much attention early during the decision-making process.

Our findings of P300 amplitudes are consistent with the behavioral performance, and the more direct evidence that supports this speculation comes from the correlational analysis between the behavioral performance and P300 amplitudes. The results showed that the greater P300 responses were associated with more response times for making decision to give the information to others. In the communal mode, people focus more on the “soft” aspects of interpersonal interaction, such as mutual support and long-term relationship, then they would concern for others rather than personal computation of costs-benefits (Mcneely, 1991; Cao et al., 2015; Wierzbicki and Zawadzka, 2016). In contrast, while giving the advice in the market mode, it elicited larger amplitudes of P300 than which in the communal mode. It suggests that compared with the communal mode, people entail more mental operation when giving advice in the market mode. The findings are supported by previous research, for example, Vohs et al. (2006) proposed that people’s self-orientation in the market mode would become more salient and thus might care less about others. Importantly, people in the market mode demand much deliberate calculative cognitive operations for their personal costs and benefits than people in the communal mode (Gasiorowska et al., 2016). Therefore, in the communal mode, the amplitudes of P300 were smaller than which in the market mode. Moreover, we found that there was no difference between the amplitudes of P300 in the communal mode and control group. In the control group, participants did not get any mental mode priming operations. Previous studies showed that advice-giving was a processing of reputation-seeking (Jonas et al., 2005; Helm and Salminen, 2010; Mobbs et al., 2015), thereby, the intuitional motivation of reputation-seeking drives them concern on others rather than thinking about their own personal costs and benefits, and the processing is consistent with the motivation and behavior of the communal mode. Reinstein and Riener (2012) proposed that an individual sent a signal of his concerns for others since improving his reputation, and this was called the processing of reputation seeking. Therefore, in the communal mode and the control group, the motivation of reputation-seeking drives people give effective information without concerning personal costs and benefits. In all, people in the market mode appear more disposed to engage in more deliberate computations involving personal costs and benefits compared with the communal mode and control group. Therefore, the amplitude of P300 in market mode was larger than the communal mode and control group.

Compared with the damnous advice, the profitable advice elicited larger P300 amplitudes in the market mode, while there was no such difference were observed in communal mode and control condition. The findings reflect that when the participants have the beneficial information, they know it will make the gain for others. They will have a calculation about personal costs and benefits, and when giving a good advice to others without any return, the cost-benefit calculation makes it more difficult to answer the question: “If I give this piece of good advice to others, why not give me the related material reward or benefits?” or “What does my beneficial advice deserve?” (Frey and Oberholzer-Gee, 1997; Korsgaard et al., 1997; Meglino and Korsgaard, 2004; Qiao et al., 2017). When giving a good advice to others, it induced a cognitive conflict in brain, thus elicited a larger amplitude of P300 compared with the poor advice. Therefore, we assume that in the market mode, the behaviors of giving the lucrative advice to others motivate more selfish concerns on personal costs and benefits compared with the poor advice.

Finally, advice-giving is a typical surrogate decision making on behalf for others in social decision making, especially in the different sociality mental modes, the motivations and orientations of the decision-making will be different. The findings of the present study are consistent with the dual-process system during decision-making (Sanfey and Chang, 2008). This system consists of two successive systems: the system 1 represents an automatic, fast, effortless, unconscious system. Moreover, system 2 is assumed to represent a controlled and effortful system implementing deliberate costs-benefits calculation. Thus, in the present study, we found the sociality mental modes had no impact on the system 1, which reflects automatic, coarse stimulus evaluation. People only processed the valence of the information based on intuitional judgment. The processing of mental modes occurred at system 2. Subsequent P300 variation indicates that processing of different mental modes entail mental resource at the later stage, which suggests that system 2 is responsible for processing the decision-making in different sociality modes.

Furthermore, the task of the present study confirms the “intentional-weighting” mechanism model (Hommel, 2009; Memelink and Hommel, 2013), which suggested that the intention-related feature dimension of the information are weighted more strongly, and values of feature defined on the dimension have a stronger impact on information processing (e.g., stimulus selection). In particular, in our study, participants were asked to select the boxes with different colors (perceptual dimensions), which have different action meanings (i.e., profitable or damnous), so that the perceptual dimensions are coded for different action feature relevant. Then the stimulus selection relies on the perceptual dimensions on which the action-relevant consequences are defined. The findings imply that values of feature defined on the dimension indeed have an impact on decision-making processing.

However, there are also some limitations in the present study. First of all, in fact, human psychology is complex within real-world situations, but the experimental situation in the present study may be too pure to consider some other social factors that might influence advice-giving. Thereby, the effects of our study may indeed be an underestimation (i.e., weaker version) of what might happen in ordinary situations (Kingstone et al., 2008; Gozli and Deng, 2017). Furthermore, culture and gender factors might also influence the effects of the present study. All participants were from China, which is living in a collectivist culture. In addition, Eagly and Steffen (1986) found that females tend to concern others more and give beneficial information to other compared with males. Thus, it would be worthwhile to consider the effect of culture and gender on advice-giving in the future.

## Conclusion

The present study has investigated the sociality mental modes effect on the processing of advice-giving by using the ERP approach. Different mental modes have different calculation of costs and benefits. Communal mode demands little mental processing and does not yields greater consistency in behavior. In contrast, the market mode depends on many more cognitive operations about costs-benefits. Our findings extend the scope of the neural mechanism of sociality mental modes modulation of advice-giving by providing a temporal description of the modulation.

## Author Contributions

JL, YlZ, and YZ designed the experiment. JL, WF, LL, and ML recruited participants and collected the data. JL, YS, and YZ performed the data analyses. JL and YZ wrote the manuscript.

## Conflict of Interest Statement

The authors declare that the research was conducted in the absence of any commercial or financial relationships that could be construed as a potential conflict of interest.
